# Emergence from general anesthesia and the sleep-manifold

**DOI:** 10.3389/fnsys.2014.00146

**Published:** 2014-08-13

**Authors:** Darren F. Hight, Vera M. Dadok, Andrew J. Szeri, Paul S. García, Logan Voss, Jamie W. Sleigh

**Affiliations:** ^1^Department of Anaesthesiology, Waikato Clinical School, University of AucklandHamilton, New Zealand; ^2^Department of Mechanical Engineering and Center for Neural Engineering and Prostheses, University of CaliforniaBerkeley, CA, USA; ^3^Department of Anesthesiology, Atlanta VA Medical Center/Emory UniversityAtlanta, GA, USA

**Keywords:** general anesthesia, emergence, sleep-manifold, connection strength, resting membrane conductivity

## Abstract

The electroencephalogram (EEG) during the re-establishment of consciousness after general anesthesia and surgery varies starkly between patients. Can the EEG during this emergence period provide a means of estimating the underlying biological processes underpinning the return of consciousness? Can we use a model to infer these biological processes from the EEG patterns? A frontal EEG was recorded from 84 patients. Ten patients were chosen for state-space analysis. Five showed archetypal emergences; which consisted of a progressive decrease in alpha power and increase peak alpha frequency before return of responsiveness. The five non-archetypal emergences showed almost no spectral EEG changes (even as the volatile general anesthetic decreased) and then an abrupt return of responsiveness. We used Bayesian methods to estimate the likelihood of an EEG pattern corresponding to the position of the patient on a 2-dimensional manifold in a state space of excitatory connection strength vs. change in intrinsic resting neuronal membrane conductivity. We could thus visualize the trajectory of each patient in the state-space during their emergence period. The patients who followed an archetypal emergence displayed a very consistent pattern; consisting of progressive increase in conductivity, and a temporary period of increased connection strength before return of responsiveness. The non-archetypal emergence trajectories remained fixed in a region of phase space characterized by a relatively high conductivity and low connection strength throughout emergence. This unexpected progressive increase in conductivity during archetypal emergence may be due to an abating of the surgical stimulus during this period. Periods of high connection strength could represent forays into dissociated consciousness, but the model suggests all patients reposition near the fold in the state space to take advantage of bi-stable cortical dynamics before transitioning to consciousness.

## Introduction

Over the last decade there has seen an increasing interest in the wake-up period following withdrawal of a general anesthetic, and in the neurobiological processes leading to the return of consciousness. We term this the emergence period; and it is defined as the time from the cessation of anesthetic delivery until the patient can make a non-reflex response to verbal command. Characterization of the changes that occur during emergence has attracted attention both from researchers searching for the neural correlates of consciousness (Mashour and Alkire, 2013) and from more clinically oriented studies, whose focus is on the quality of recovery of patients following surgery (e.g., Law et al., 2011). Much of this interest has come from the realization that the induction process (the entrance to the anesthetized state) and emergence process (the exit from the anesthetized state) are not simply the mirror image of each other, but rather that emergence is an active process characterized by a distinct neurobiology (Kelz et al., 2008; Lee et al., 2011; Kushikata and Hirota, 2014). For example, the time required for the return to consciousness shows a much greater variability than the time required for the loss of consciousness. These distinct processes occur at different drug concentrations—a classic hysteresis effect (Friedman et al., 2010). This effect, traditionally considered a pharmacokinetic “artifact” arising from a delayed and variable rate of removal of the an anesthetic agent from brain sites, may in fact be due to a biological tendency of the central nervous system to resist transitions between conscious and unconscious states. The emergence period is also of critical importance from a clinical perspective. For example, during emergence patients may infrequently face life-threatening complications (Kushikata and Hirota, 2014), but more often can wake up experiencing high levels of pain and nausea (Law et al., 2011), despite pre-emptive analgesia. Patients can also experience periods of confusion and disorientation, or even delirium following wakeup, indicating a possible incomplete return of full consciousness. The incidence of emergence delirium in children has been reported as high as 50% in preschool children (Banchs and Lerman, 2014), but the mechanisms of this phenomenon are not well understood. Clinicians often note the huge variability in wakeup length and quality, but find the prediction of quality of recovery from the intraoperative period a major challenge.

### EEG and behavioral changes during emergence

The most practical non-invasive method for observing state changes in brain function during anesthesia is the electroencephalogram (EEG). Changes in the EEG have been observed in anesthetized patients since 1937 (Gibbs et al., 1937). At the deepest levels of anesthesia EEG activity is suppressed (isoelectric) for short periods before returning to bursts of activity. This pattern, labeled burst suppression, is not seen in natural sleep. At levels of anesthesia required for surgery the amplitude of the EEG is often large in the delta (1–4 Hz) and alpha (8–14 Hz) ranges, showing a similar but distinct waveform to that of slow-wave natural sleep (see Brown et al., 2010 for a review). For a more detailed treatment of the EEG during anesthesia see (Bennett et al., 2009). Following the end of surgery, when the anesthetic is turned off, it is commonly believed that the delta and/or alpha dominated waveform disappears, being replaced with a beta (15–30 Hz) waveform just prior to waking. For some patients showing alpha dominated waveforms, the alpha activity increases in frequency by about 2–3 Hz during emergence, as observed by Purdon et al. (2013). The topography of the EEG also changes during emergence, with the frontally dominant (coherent) alpha activity during anesthesia shifting to occipital areas before the patient awakes (Gugino et al., 2001). Clinically, a progression can often be seen in the patient's responses that loosely correlate with these changes in EEG pattern. These observations include a return in spontaneous respiration, followed by brainstem responses such as salivation and tearing and gagging on the endotracheal tube, if still in place. This is then followed by non-purposive or defensive movements (with eyes remaining closed), before the patient can finally respond to a command (Brown et al., 2010; Langsjo et al., 2012).

Despite a burgeoning number of anesthesia-related EEG studies in recent years, we know of only two recent research groups who have looked at the EEG in detail during the emergence period, as shown in the articles from Purdon et al. (2013) and Lee et al. (2011). Research from the group based in Massachusetts General Hospital (Purdon et al., 2013) has focused on the characteristics of the EEG during propofol anesthesia while measuring responsiveness with an auditory stimulus consisting of a tone or the patient's name being delivered to the subject every 4 s. These researchers used a 64 electrode multichannel EEG system during a slow propofol induction and subsequent emergence. When they analyzed the EEG from a temporal-spectral perspective, one of the key characteristics they noted was an increase in median frequency of the alpha band of the EEG during the return to consciousness. The authors named this increase in frequency the “traveling peak” to emphasize the continuous nature of the change in the frequency domain, an observation which is obscured if analyzing the power of the EEG in traditional separate frequency bands. From a phase-amplitude perspective, they also observed coupling between the phase of slow-wave oscillations (0.1–1 Hz) and the amplitude of the alpha (8–14 Hz) band; noting that in deep anesthesia the alpha amplitudes were highest when the slow oscillation was also highest, calling this “peak-max.” During the transition to consciousness this phase relationship reversed so that the largest amplitude alpha activity occurred at the lower values of the slow oscillation, or “trough-max.” Purdon and colleagues (Purdon et al., 2013) also looked at spatial coherence and reported that, at the return to consciousness, coherent spatial activity shifts from frontal to occipital regions. These researchers have been looking for a few spectral features that can reliably track anesthetic depth and the return of consciousness, and they concluded that the emergence from a propofol anesthetic was marked by a gradual transition to consciousness, the level of which is dependent on stimulus saliency—emotional or neutral auditory input.

In contrast, Lee et al. (2011) examined the network properties of the anesthetic state during emergence, using cross-correlations of the EEG in multiple channels to estimate cortical connectivity, and a novel method to account for genuine versus spurious levels of connectivity. Two different patterns of changes in connectivity strength and topography on awakening were noted, the first where the increase in connection strength was abrupt on wakeup, and the second where connection strength showed a gradual change on awakening. Subjects were then categorized into these categories for further analysis. They concluded that there were likely multiple pathways of return to consciousness, which one single theory of anesthesia would not be able to explain.

In both of the aforementioned studies, *none* of the subjects were undergoing surgery and all were healthy volunteers. However general anesthesia is administered in order to allow surgery to take place. Do EEG's recorded in the clinical context show the same patterns during emergence as those recorded from healthy volunteers, without any surgical noxious stimuli? Is there one common pathway to responsiveness or are there multiple pathways?

### Linking molecular level actions to EEG and behavior in anesthesia

One of the drawbacks of the EEG is that, at best, it provides a somewhat opaque window into the underlying mechanisms governing anesthesia state changes in the brain. Thus, despite a well-advanced understanding of the molecular level mechanisms of most anesthetics (see Brown et al., 2011 or Alkire et al., 2008 for reviews), there is a gap in understanding as to how these molecular mechanisms link with the EEG patterns and associated changes in consciousness. One accepted method for attempting to bridge this gap is the use of EEG modeling of anesthesia. Here the goal is to replicate features of the clinically observed EEG with the output signal from a model which has a biologically realistic set of parameter constraints. What follows is a very short overview of types of anesthesia modeling, linking proposed molecular and neural mechanisms of anesthetic action to the structure of the EEG in anesthesia.

The previously mentioned research group (Purdon et al., 2013) have recently published a thalamocortical model as an explanation for the EEG alpha rhythm that is seen in propofol anesthesia (Ching et al., 2010). This model exemplifies the neural-biophysical approach (Ching and Brown, 2014), and builds on an earlier cortical networks model developed by McCarthy et al. (2008). In this model a network of cortical pyramidal neurons with associated interneurons are coupled to the thalamus. The action of propofol is modeled as an increase in the conductance and decay time of the GABA_A_ inhibitory current, which, based on earlier work proposing mechanisms for thalamic alpha activity (Contreras et al., 1997), leads to an entrainment of oscillations between the thalamus and cortex. These alpha oscillations then become visible in the frontal cortex, and in the model as the summed activity of the pyramidal neurons—a surrogate for the EEG. A spectrogram is then used to compare features of the model output to the known effect of propofol on the EEG.

Another form of modeling, the mean-field method, describes the mesoscale population activity resulting from short and long range interactions between sets of inhibitory and excitatory neurons. The advantages of this approach are that it is possible to use physiologically plausible parameter values, and that the output of the model can be related to the local field potentials and hence can be directly compared with the experimentally-obtained EEG or ECoG electrode output. Because of the averaging involved in the mean-field models, they are much less computer resource intensive than neuron-by-neuron simulations, and thus are more tractable for phenomena that involve cortical activity at larger length scales. They also have the advantage that they are often simple enough to allow the application of classical mathematical analytic methods, rather than being mere simulations. However such models do not include much specific neuroanatomy, and hence are mainly used to model widespread global central nervous system disturbances such as sleep, and seizures. The application of these methods to anesthesia modeling was first described by Steyn-Ross and Liley (Steyn-Ross et al., 1999). At its heart, this model describes the time evolution of the mean soma potential in populations of interacting inhibitory and excitatory cortical neurons. The authors modeled the propofol effect as an increase in the area under the curve of the inhibitory post synaptic potential; and found that increasing anesthetic concentration could lead to multiple stable dynamical states, and that the sudden phase transition between these states mimicked that observed in the EEG and level of consciousness during induction of anesthesia. Subsequently there has been a steady stream of papers that have looked at a variety of questions relating to various mechanisms of sleep, anesthesia, and seizures (e.g., Bojak and Liley, 2005; Sleigh et al., 2009; Hutt and Longtin, 2010; Ching et al., 2012; Liley and Walsh, 2013). We refer the reader to an excellent recent review of general anesthesia models by Foster et al. (2008). However the comparison between the “pseudo-EEG” output from the model, and the real EEG obtained from experiments, has always been semi-quantitative at best. Therefore, using the Steyn-Ross model as a basis, following Lopour et al. (2011), Dadok et al. (2014) developed Bayesian methods to solve the inverse problem of mapping experimentally derived EEG features data back onto the state-space of the model. In this way the association of specific values of model parameters corresponding to each epoch of real EEG might give insight into the underlying neurobiology. It is in some ways similar to the dynamic causal modeling approach (Marreiros et al., 2010), but is subject to more realistic neurobiological constraints. Working from sleep EEG data, they explored the statistical usefulness of combinations of various EEG “features” through which an association could be made to a specific set of parameter values in the model, and hence probabilistically estimate how the neurobiological parameters might change with time, mimicking what is happening within a patient's brain. Typically this is displayed as a progression, tracking a path on a 2-dimensional parameter manifold. In this way they successfully showed that progressive cycles of natural sleep could be displayed as a continuous trajectory on a sleep manifold (Dadok et al., 2014). This probabilistic method has never been applied to anesthesia or to the emergence period. In this study we aim to apply the method of Dadok et al. (2014) to EEG recorded during anesthesia, and over the emergence period. Specifically we wanted to answer the questions: do patients show homogenous emergence EEG patterns? Do these EEG patterns suggest different underlying biological processes? Can we use the model to infer these biological processes from the EEG patterns?

## Methods

### Emergence period recordings

Eight-four patients (40 females) aged between 21 and 88 (average age, 61 years) with an American Society of Anesthesiologists (ASA) physical status between I and IV having surgery at the Waikato District Health Board Hospital, Hamilton, New Zealand, were recruited for this study. Two cases were rejected due to faulty or absent EEG recordings. All participants gave informed consent and the study was approved by the New Zealand Health and Disability Ethics Committee (Ref. 12/CEN/56). EEG waveforms were recorded from the forehead (location Fpz on the 10/20 montage) via single-use electrode strips using either the Bispectral Index^®^ (BIS^®^; Aspect Medical Systems, Newton, MA, USA) or Entropy (GE Healthcare, Helsinki, Finland) depth of anesthesia monitoring systems. Other routine monitoring data [such as heart rate, blood pressure, and end-tidal volatile gas anesthetic (VGA) concentrations] were recorded from the S/5 Anesthesia Monitor (GE Healthcare, Helsinki, Finland) using the S/5 Collect program provided by the same company. Delivery and dose of opioid analgesics were also recorded during and following the operation. No restrictions were placed on anesthetic conduct during the surgery. Following the operation, the time of cessation of anesthetic delivery was noted, this time point being the start of the emergence period. A standard low-stimulus emergence protocol was followed. After oropharyngeal suction the patient was not stimulated until MAC <0.1, then they were given a series of verbal commands at 30 s intervals. The end of the emergence period was counted as the moment the patient spontaneously opened their eyes for more than 5 s, clearly engaging with the environment, or could respond to the command “Open your eyes!” (ROR). In the Post-Anesthetic Care Unit (PACU) patients were asked to give a verbal pain-score ranging from 0 (no pain) to 10 (worst pain imaginable) on awakening, and at 15 and 30 min after awakening. Ramsay Sedation scores, observed distress and instances of nausea and vomiting were also recorded.

### EEG data collection and processing

The raw EEG signal and monitoring data were analyzed with custom Matlab software (The MathWorks, *Inc.*, Natick, MA, USA). EEG waveforms were down-sampled to 100 Hz and were low-pass filtered using a Butterworth, non-aliasing low-pass (48.5 Hz, 3rd order) filter, to remove the 50 Hz artifact. Monitoring data were interpolated from every 5 or 10 s to one sample per second. From end-tidal VGA concentrations, brain effect-site concentrations (C_e_MAC) were derived from age-adjusted MAC values (Nickalls and Mapelson, 2003) assuming a T1/2Keo of 150 s. After the patient was moved to PACU, the C_e_MAC were extrapolated out until end of emergence using a decaying exponential. Opioid effect-site concentrations were converted to Fentanyl-equivalent estimates (C_e_Fentanyl) using a Fentanyl to Morphine efficacy ratio of 20:1. Opioid effect site concentrations were estimated using pharmacokinetic modeling based on population derived parameters. Thus the actual drug concentrations for each individual patient might be expected to lie within approximately a two-fold range.

**Figure 1:**
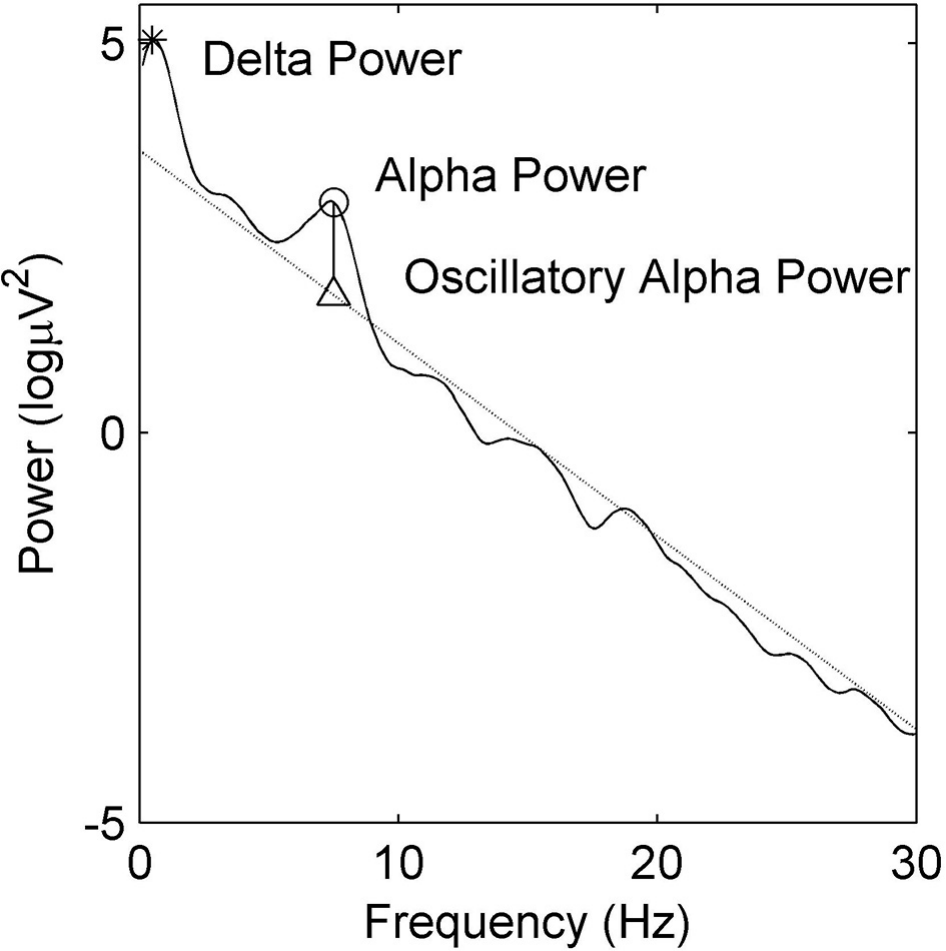
**Example power spectral density estimate showing absolute delta (∗) and alpha (◦) power**. Oscillatory alpha power is the difference between maximal alpha power (◦) and the alpha power at the linear regression estimate (△).

The power spectrum (in dB) of the EEG was calculated using the multi-taper Chronux method (www.chronux.org, Mitra and Bokil, 2008) with a time-bandwidth product *TW* = 4, and *K* = 7 tapers. We used a moving window of 4 s with an overlap of 3 s in the creation of the spectrograms. Spectrograms were taken of the observation period, which included a 15 min window prior to the emergence period; the emergence period itself began at the time of shutting off the VGA and increasing the fresh gas flows in order to flush out anesthetic agent (plotted as a vertical green line) and ended with ROR (plotted as a vertical red line). For each window the local regression fitting and likelihood method of smoothing from Loader (1997), (Locfit, included in the Chronux package for Matlab) was fitted to the power spectrum (bandwidth parameter, *h* = 1.5 Hz). We particularly noted the alpha (8–16 Hz) power and frequency, the delta power (1–4 Hz), and the presence of obvious oscillation in other frequencies [beta (16–32 Hz) and theta (4–7 Hz)]. We quantitatively obtained the maximum alpha frequency (allowable range for alpha peak was between 7 and 17 Hz), and the magnitude of both the oscillatory alpha-power above the underlying broadband noise (Leslie et al., 2009, see our Figure 1) and the delta power. It must be noted that patients who wake after surgery often are somewhat disoriented, and thus the large amounts of electromyographic (EMG) activity make it difficult to quantitatively interpret the EEG signal after return of responsiveness to verbal command (ROR).

### Sleep-manifold modelling

The primary aim of this study was to examine the different biological mechanisms underlying the observed EEG changes. Because the wide variability observed between different patients during emergence will obscure the EEG changes that may be seen within each patient, we explicitly chose not to average all the results, but instead to investigate the emergence trajectories of 10 representative patients in detail. From our data-set, we chose five EEG recordings that were typical examples of gradual transitions in waveform spectral power over emergence, similar to those seen in Purdon et al. (2013). Based on previous literature, we have termed this the “archetypal” emergence trajectory. As counter-examples, we also chose another five recordings which showed no notable transition in EEG spectral power prior to abruptly awakening (i.e., our non-archetypal wakeups). We anticipate that the methodology developed in this paper might be used in subsequently studies with large enough numbers of patients to enable accurate statistical estimation methods. Thus 10 EEG recordings for the observation period were used as input into the sleep manifold model of Dadok et al. (2014). More detailed method descriptions are available in that paper, but in short, the model consists of a set of partial differential equations that describe the time evolution of the mean soma potentials of a two-dimensional homogeneous system of coupled inhibitory and excitatory neurons, representing a macrocolumn (about 100,000 neurons) of undifferentiated association cortex. This corresponds to the size of typical excitatory neuronal dendritic arborization. Each macrocolumn also receives excitatory input from surrounding cortex and nonspecific white noise input from subcortical structures. We only consider fast chemical synaptic inputs [mediated via gamma-amino-butyric acid (GABA) and α-amino-3-hydroxy-5-methyl-4-isoxazolepropionic acid (AMPA) channels]. The model parameters are chosen based on experimentally measured biological values; and include the magnitude and duration of excitatory and inhibitory synaptic potentials, the effects of reversal potentials, the form of the sigmoid relationship between probability of firing and soma potential, and the effect of leak currents on the resting membrane potential. We have chosen to describe the model output and the experimental results based on a 2-dimensional state space using the change in resting membrane impedance (Δh^rest^_e_) and the cortical excitatory synaptic strength (L) as the axes. These were chosen because:

- The Δh^rest^_e_ is a measure of the neuronal membrane impedance. This is the inverse of conductivity, and is largely controlled by the intrinsic neuronal currents (particularly the potassium currents). These currents, in turn, are inhibited by subcortically driven aminergic and cholinergic arousal neuromodulators. Thus the conductivity may be seen as an indication of the balance between suppression and arousal—as mediated by brain stem modulation of the cortex, similar to that found in natural sleep-wake states.
- The excitatory strength is an indicator of synaptic connectivity between cortical pyramidal neurons. This might be seen as a direct index of how the anesthesia directly disrupts a cortical functionality that can be directly linked to known EEG indices of regional connectivity.

**Figure 2:**
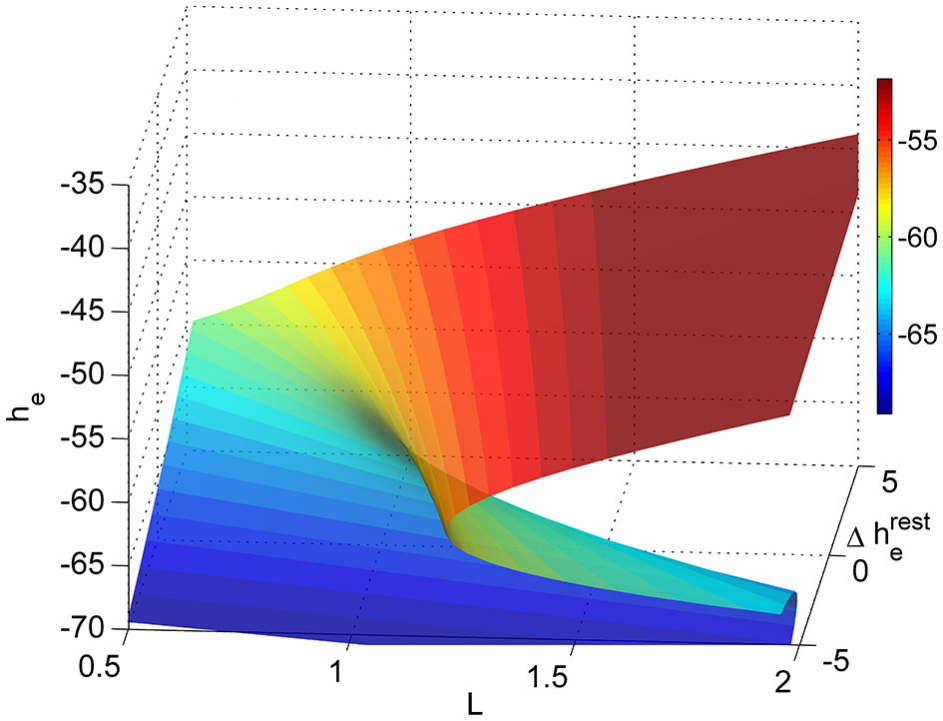
**Sleep-manifold showing the folding state-space surface (folded area is bi-stable) over a two dimensional parameter space (adapted from Dadok et al., 2014)**.

Thus we attempted to somewhat separate these two known components of VGA action. As described in Dadok et al. (2014) we then solve the equations to produce a sheet manifold of steady states. The manifold is shown in Figure 2. It can be seen that, at low values of L and Δh^rest^_e_ the resting steady state is relatively hyperpolarized (the blue area in the left lower region); as L and Δh^rest^_e_ increases the steady states become more depolarized (red). However there is a region in which there are three steady states (two stable and one unstable)—the fold in the manifold. This area is within the bold black inverted “Y” region in subsequent figures. Around this area there is the possibility for the model brain to jump discontinuously between low firing and high firing modes. At each point on the manifold there are fluctuations in soma potential that produces a “pseudoEEG” for that point in state space.

In brief the method of probabilistically mapping experimental data to the model manifold is as follows:

(i) “Features” are extracted from sequential 30 s segments of the raw EEG.

These features are derived indices that are felt to contain the important dynamic information contained in the raw EEG. The process of choosing features is complex and inherently heuristic. We used the same features as had been found most useful for natural sleep; namely the slope of the power spectral density, the spindle index, combination delta wave steepness (the mean delta gradient), and the equiprobable mutual information (Dadok et al., 2014).

(ii) The magnitude of each feature is mapped onto the state-space manifold of the model.

We used the same state-space manifold as used to describe natural sleep. The choice of these parameters is arbitrary to some degree, but they were chosen to reflect information about two relevant facets of neuronal function—namely synaptic efficiency and intrinsic neuronal currents.

(iii) For each real EEG segment, the probability (log-likelihood) of its associated 4-dimensional feature vector is mapped onto the state-space manifold, using a naïve Bayesian algorithm.

This procedure allows the unbiased determination of what regions of state-space are likely to be associated with any particular EEG pattern. Again this has been optimized for natural sleep, as we are able to compare the model results with established sleep scoring methods.

(iv) The temporal evolution on the state-space manifold through the course of emergence is shown by the trajectory of the probability centroid for each segment.

The trajectory thus acts as a link to indicate how changes in the scalp EEG might reflect the cortical neuronal function as the patient emerges from anesthesia. At present it is not known if this methodology is robust to EEG noise and to the choice of different model parameters.

## Results

### Variance in alpha activity prior to emergence

**Figure 3:**
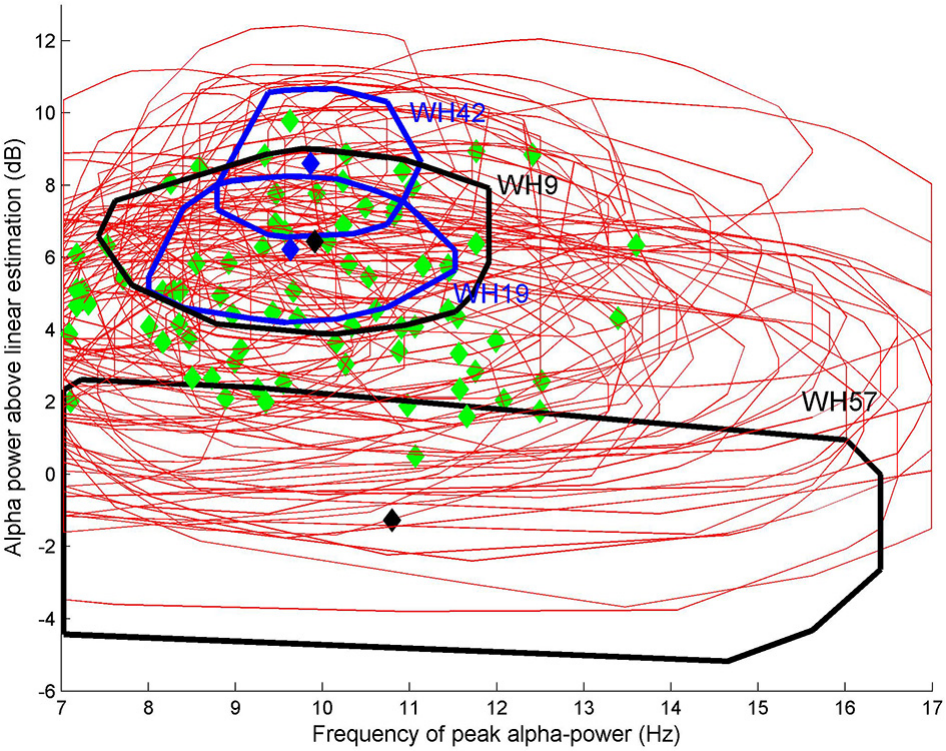
**Oscillatory alpha power against alpha frequency for each of 82 patients for the 15 min prior to the start of emergence**. Green diamonds are the centroid and red ellipsoids the area of 80% of the closest points from the centroid. Four patients are displayed in either blue (WH19 and WH42, “archetypal” transitions) or black (WH9 and WH57, “non-archetypal” transition).

Patients emerge from general anesthesia differently. As an example we show the alpha frequency and power for all 82 patients for the 15 min prior to the start of emergence in Figure 3. The green diamonds are the centroid and the red ellipsoids the area of 80 percent of the closest points from the centroid. This figure demonstrates that there is a wide variation in alpha-power and frequency both between and within patients. Four of the ten patients of interest are displayed in blue (WH19 and WH42, gradual “archetypal” transitions in EEG waveform over the emergence period) and black (WH9 and WH57, “non-archetypal” transitions in EEG during emergence period). All of these four patients emerged from a sevoflurane anesthesia, except for patient WH9 who had a desflurane anesthesia. We now describe, in more detail, the changes in EEG, drug concentrations, and putative modeled changes in biological parameters during emergence period for these four examples—two archetypal and two non-archetypal.

### Patient WH19: archetypal emergence (alpha frequency increase, alpha loss, delta loss)

**Figure 4:**
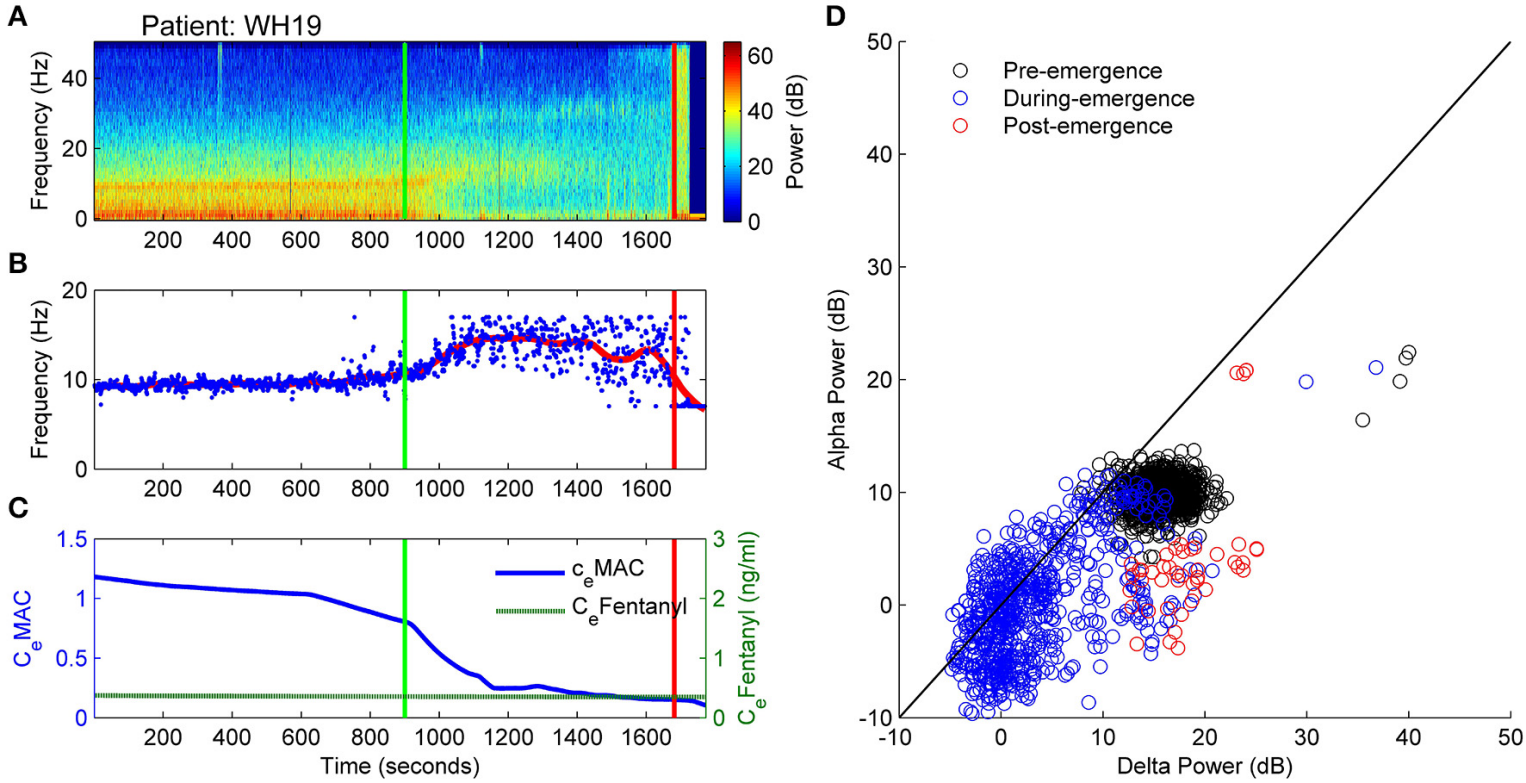
**Patient WH19: Archetypal emergence (alpha frequency increase, alpha loss, delta loss). (A)** Spectrogram of the observation period. Start of emergence shown as a vertical green line at 900 s, time of patient response as a vertical red line. **(B)** Frequency of maximal oscillatory alpha power. **(C)** Concentration of anesthetic gas (C_e_MAC), blue line, left vertical axis, and opioid levels as equivalent Fentanyl (C_e_Fentanyl, ng/ml), green line, right vertical axis. **(D)** Absolute alpha power (dB) against absolute delta power (dB) for prior to start of emergence (black circles), during emergence period (blue circles), and following recovery of response (ROR).

In Figure 4 the spectrogram of the observation period (Figure 4A) for patient WH19 is shown from 15 min prior to start of emergence (vertical green line at 900 s) until after patient response (ROR, vertical red line). Prior to start of emergence it shows clear bands of alpha (centered at 10 Hz as seen in Figure 4B) and delta activity. Over this period (until 900 seconds) anesthetic concentration had decreased slightly from 1.2 to 0.8 C_e_MAC, while opioid levels remained low (C_e_Fentanyl 0.2 ng/ml) over the whole observation period (Figure 4C). Following start of emergence the frequency of maximal alpha power is seen to increase by 2–3 Hz (Figure 4B) and then disappearing several min before ROR. A band of beta activity centered at 30 Hz can also be seen beginning at around 1100 s and continuing until ROR. From 1000 s onwards the alpha band in the spectrogram (Figure 4A) became smaller and there was an increase in spread of frequencies in Figure 4B, so that there was more variation in the detection of the alpha oscillatory peak. In Figure 4D it can be seen that the pre-emergence waveform (black circles) is tightly constrained, maintaining uniform levels of alpha and delta power. During emergence (blue circles) a clear progressive decrease in both alpha and delta power is seen, decreasing to a level of 0 dB for both bands. This absence of both alpha and delta power can also be seen clearly in the spectrogram (Figure 4A). After emergence, delta power increases (red circles in Figure 4D), as does power in all frequency bands (seen in the orange vertical band following the vertical red-line at around 1700 seconds in Figure 4A).

**Figure 5:**
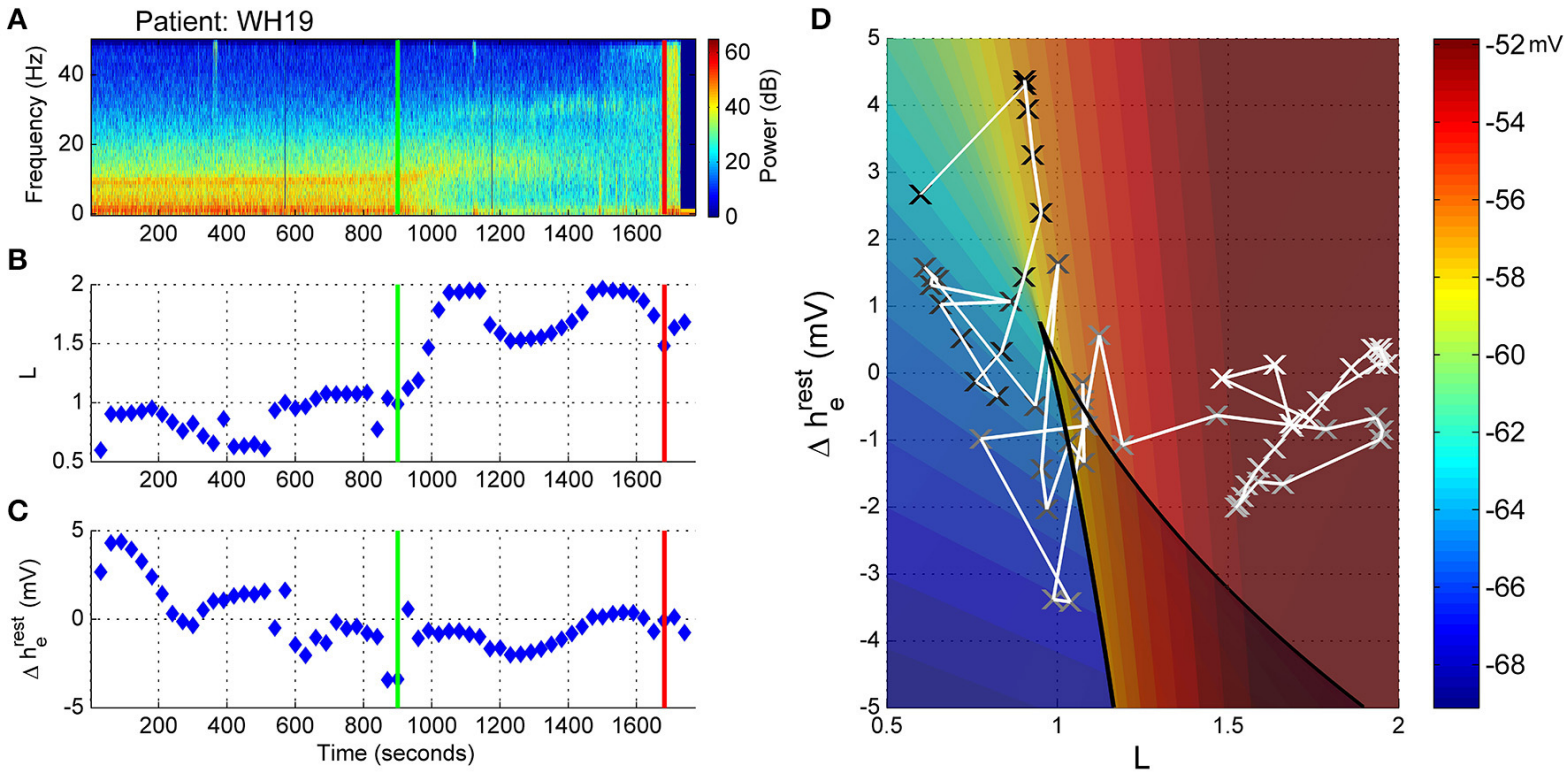
**Patient WH19, Sleep Manifold. (A)** Spectrogram as in Figure 4. **(B)** Excitatory connection strength (L-parameter) over period of observation. **(C)** Change in resting membrane impedance (Δh^rest^_e_) over period of observation. **(D)** Resultant positioning on the sleep-manifold, with a black cross being the start of, and a white cross being the end of the observation period, with intermediate shades on the gray-scale representing the time progression between these time-points.

How do these EEG alterations translate into changes in the underlying model (and perhaps brain) parameters? Figure 5D depicts the progression in L and Δh^rest^_e_ parameters on the 2D sleep-manifold over the same time-period—with a black cross being the start and a white cross being the end, and intermediate shades on the gray-scale representing the progression between these time-points. In Figure 5 excitatory connection strength (parameter L, shown in Figure 5B) remained at low levels prior to emergence, and doubled after the start of emergence. The neuronal membrane impedance (Δh^rest^_e_ in Figure 5C) progressively decreased in the period before emergence had even started. The resultant trajectory occupies two regions on the sleep-manifold. In the first phase, which corresponds to the time before the start of emergence, there was a large decrease in resting membrane impedance with little change in connection strength, resulting in a migration down the sleep-manifold. The reasons for this are not clear, but may be related to the small changes in C_e_MAC or decreasing surgical stimulus. The second phase, corresponding to the emergence period, showed a sharp increase in connection strength with minimal change in resting impedance, resulting in a jump to a new parameter state-space position on the higher branch of the sleep-manifold before jumping back to the lower branch before ROR (Figure 5D)—apparently entering a preliminary period of high firing without successfully achieving responsiveness.

### Patient WH42: archetypal emergence. (alpha frequency increase, alpha loss, persistent delta)

**Figure 6:**
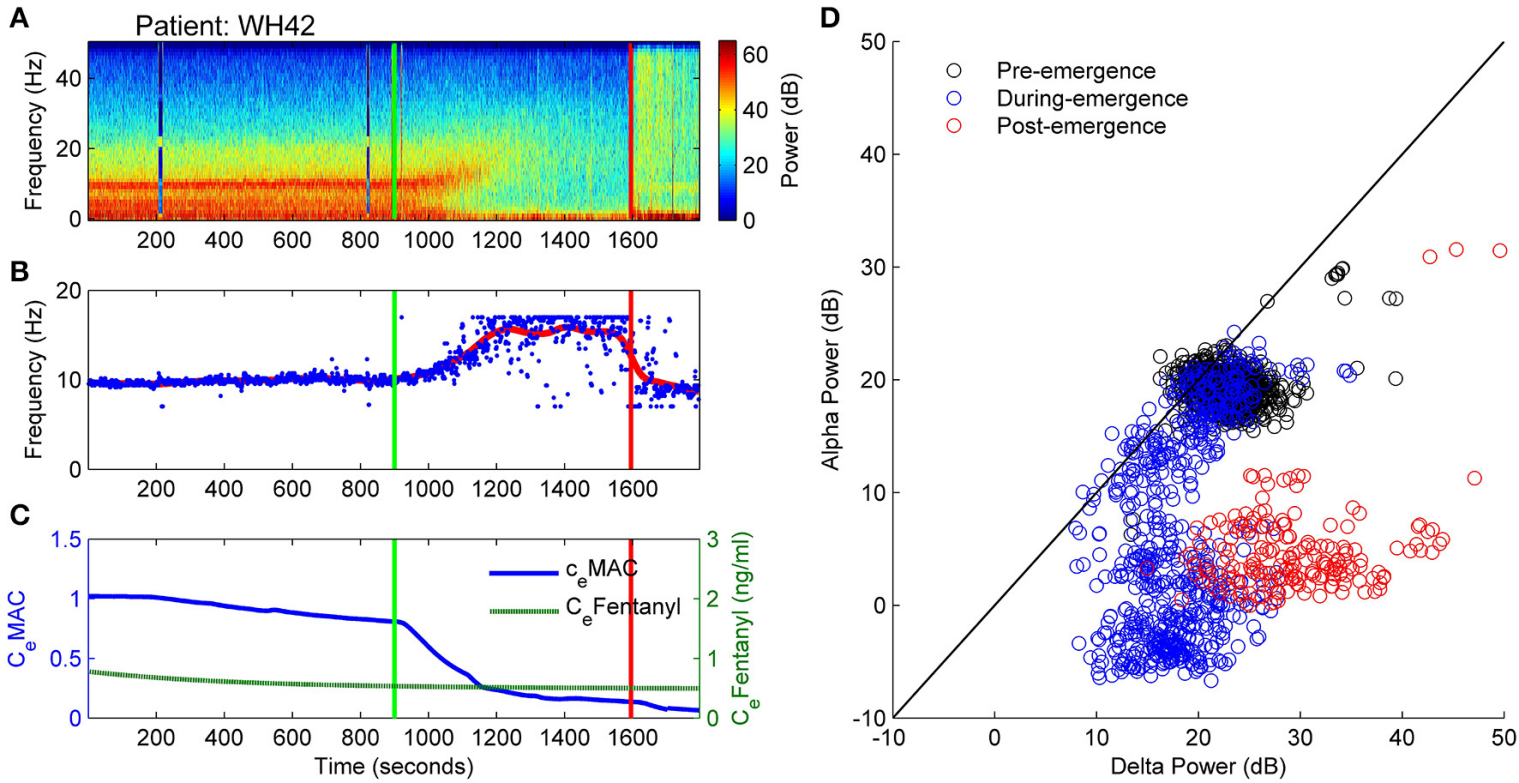
**Patient WH42**. Archetypal emergence. (alpha frequency increase, alpha loss, persistent delta). **(A)** Spectrogram of the observation period. Start of emergence shown as a vertical green line at 900 s, time of patient response as a vertical red line. **(B)** Frequency of maximal oscillatory alpha power. **(C)** Concentration of anesthetic gas (C_e_MAC), blue line, left vertical axis, and opioid levels as equivalent Fentanyl (C_e_Fentanyl, ng/ml), green line, right vertical axis. **(D)** Absolute alpha power (dB) against absolute delta power (dB) for prior to start of emergence (black circles), during emergence period (blue circles), and following recovery of response (ROR).

The spectrogram of Patient 42 (Figure 6A) is similar to that of patient WH19 above in that a clear alpha and delta band is seen prior to start of emergence, with the alpha activity increasing, before disappearing following the start of emergence (Figure 6B). However, patient WH42 starts with stronger alpha and delta power and does not lose the delta power at all prior to ROR. C_e_Fentanyl levels were also quite low [0.7–0.5 ng/ml over the observation period (Figure 6C)]. Figure 6D is also similar to that of patient WH19 in Figure 4D in that the absolute alpha and delta power are uniform prior to emergence (black circles), and show a decreasing alpha power over emergence (blue circles). In contrast to WH19 delta power remains high at around 15 dB even during the later phases of emergence. After emergence the broad band high power is caused by EMG activation and movement.

**Figure 7:**
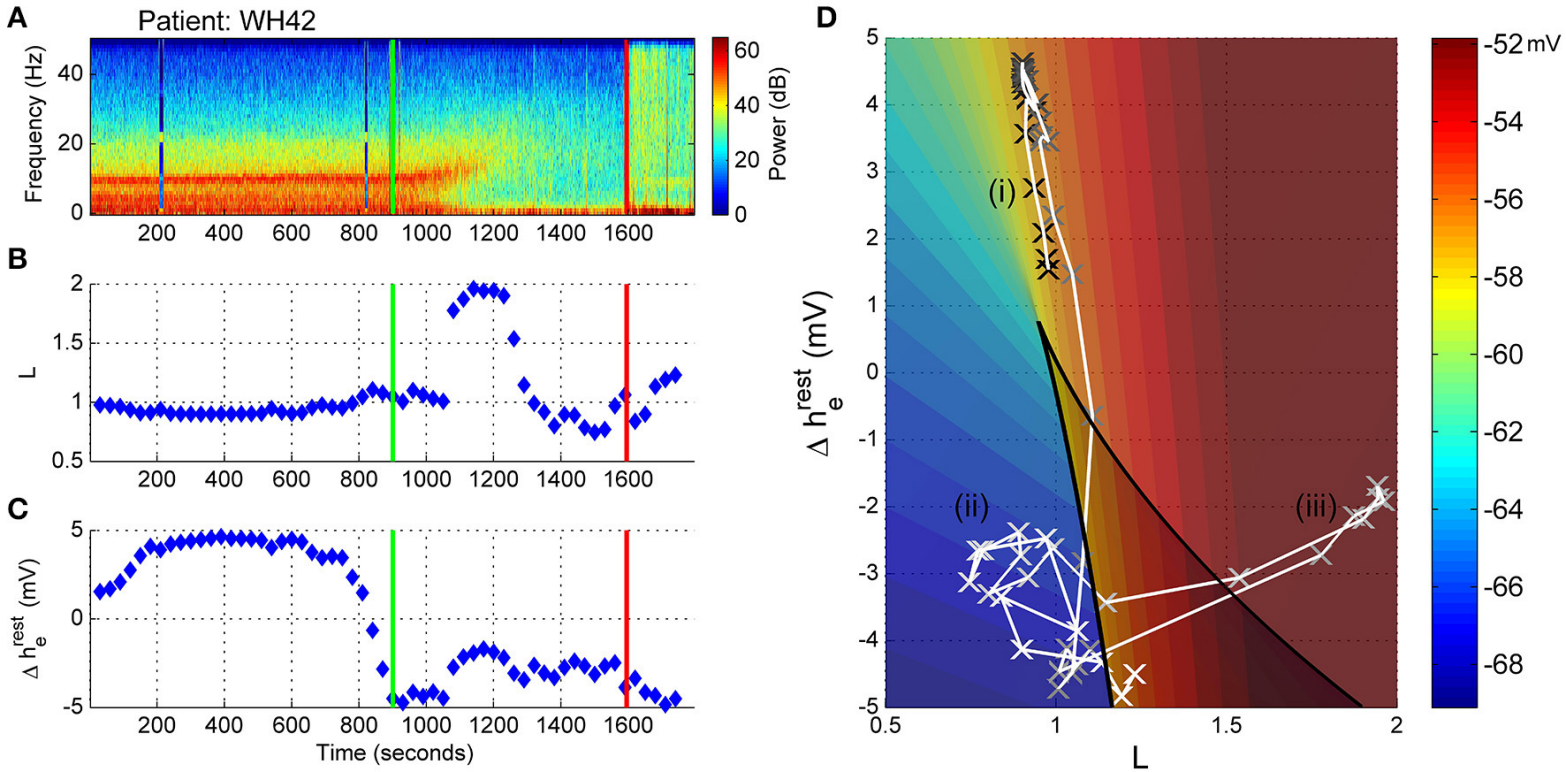
**Patient WH42, Sleep-Manifold. (A)** Spectrogram as in Figure 6. **(B)** Excitatory connection strength (L-parameter) over period of observation. **(C)** Change in resting membrane impedance (Δh^rest^_e_) over period of observation. **(D)** Resultant positioning on the sleep-manifold, with a black cross being the start of, and a white cross being the end of the observation period, with intermediate shades on the gray-scale representing the time progression between these time-points.

Similar to WH19, patient WH42 has a low connection strength value (L parameter) prior to start of emergence. As the alpha power decreases in the emergence period (Figure 7A), the connection strength rapidly increases to a value of nearly 2.0 from 1100 to 1300 s (Figure 7B). For the remaining part of the emergence period connection strength falls again to values between 0.7 and 1.1. Presumably the algorithm is being controlled by the persistent strong delta power in this phase. The change in resting impedance Δh^rest^_e_ parameter in Figure 7C is high for most of the period prior to start of emergence, but quickly drops to −5 mV just prior to start of emergence and remains low for the emergence period. When seen on the sleep-manifold (Figure 7D), the patient appears to move between three distinct parameter attractors, corresponding to: (i) prior to start of emergence (ii) early and late sections of the emergence period, and (iii) a short period of high connection strength in the middle of the emergence period, similar to the previous patient. This patient mentioned they had been having very realistic dreams before awakening.

### Patient WH9: non-archetypal emergence: minimal alpha loss, persistent theta and delta

**Figure 8:**
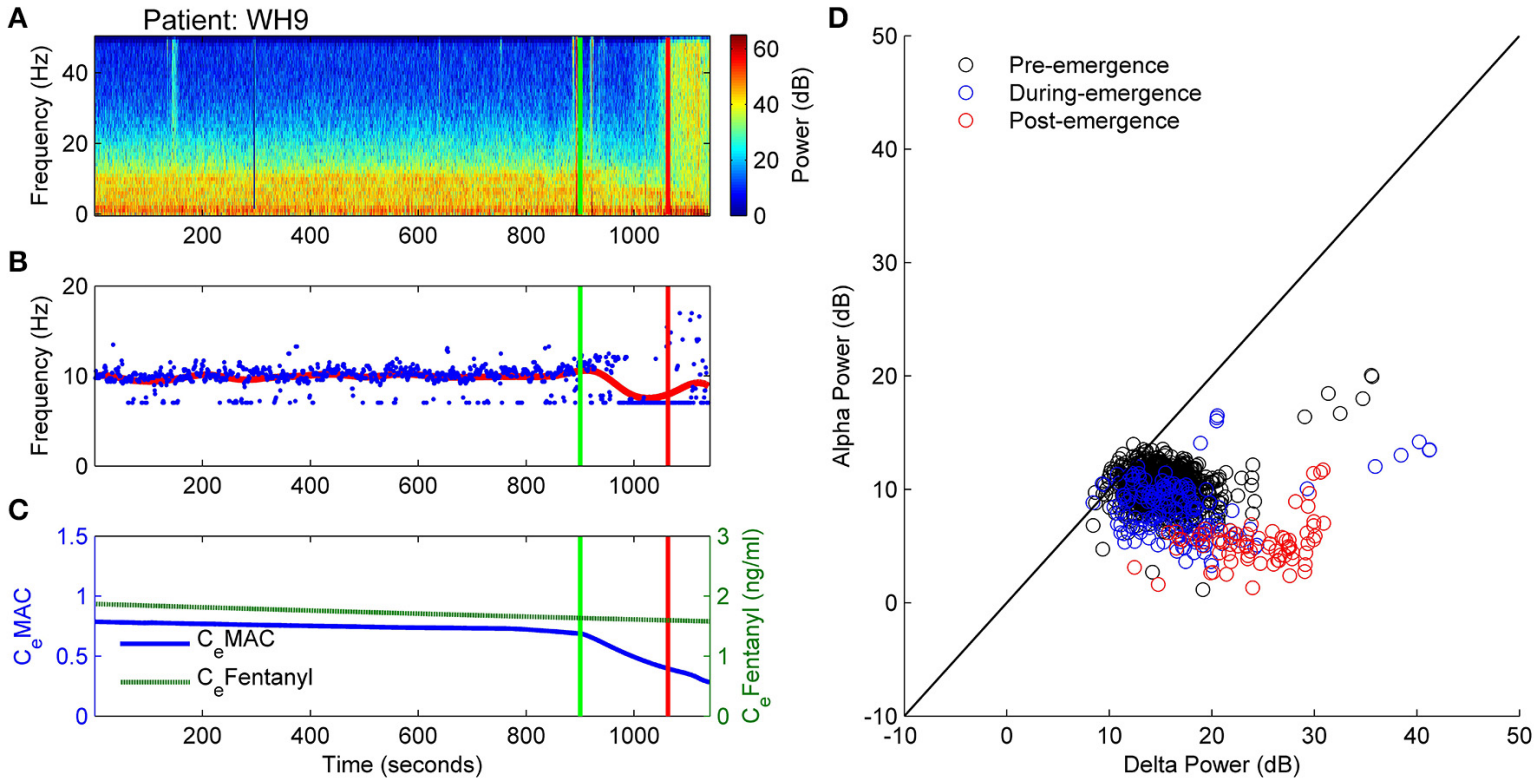
**Patient WH9: Non-archetypal emergence: minimal alpha loss, persistent theta and delta. (A)** Spectrogram of the observation period. Start of emergence shown as a vertical green line at 900 s, time of patient response as a vertical red line. **(B)** Frequency of maximal oscillatory alpha power. **(C)** Concentration of anesthetic gas (C_e_MAC), blue line, left vertical axis, and opioid levels as equivalent Fentanyl (C_e_Fentanyl, ng/ml), green line, right vertical axis. **(D)** Absolute alpha power (dB) against absolute delta power (dB) for prior to start of emergence (black circles), during emergence period (blue circles), and following recovery of response (ROR).

This patient showed no warning of imminent ROR. Figure 8A displays the spectrogram for patient WH9. Power was concentrated in bands of waveform activity corresponding to the alpha, theta and delta bands. In contrast to the previously described archetypal patterns, there was absolutely no change in power in any of these bands until ROR—with the exception of a decrease in 10 Hz alpha from about 950 s, as seen in Figure 8D, and also shown in Figure 8B as artefactual detection of the theta band at the lower limit of 7 Hz. After emergence, high levels of power were distributed evenly over the frequency spectrum, indicating the return of muscle activity artifact. C_e_Fentanyl was relatively high (1.5–2 ng/ml) (Figure 8C).

**Figure 9:**
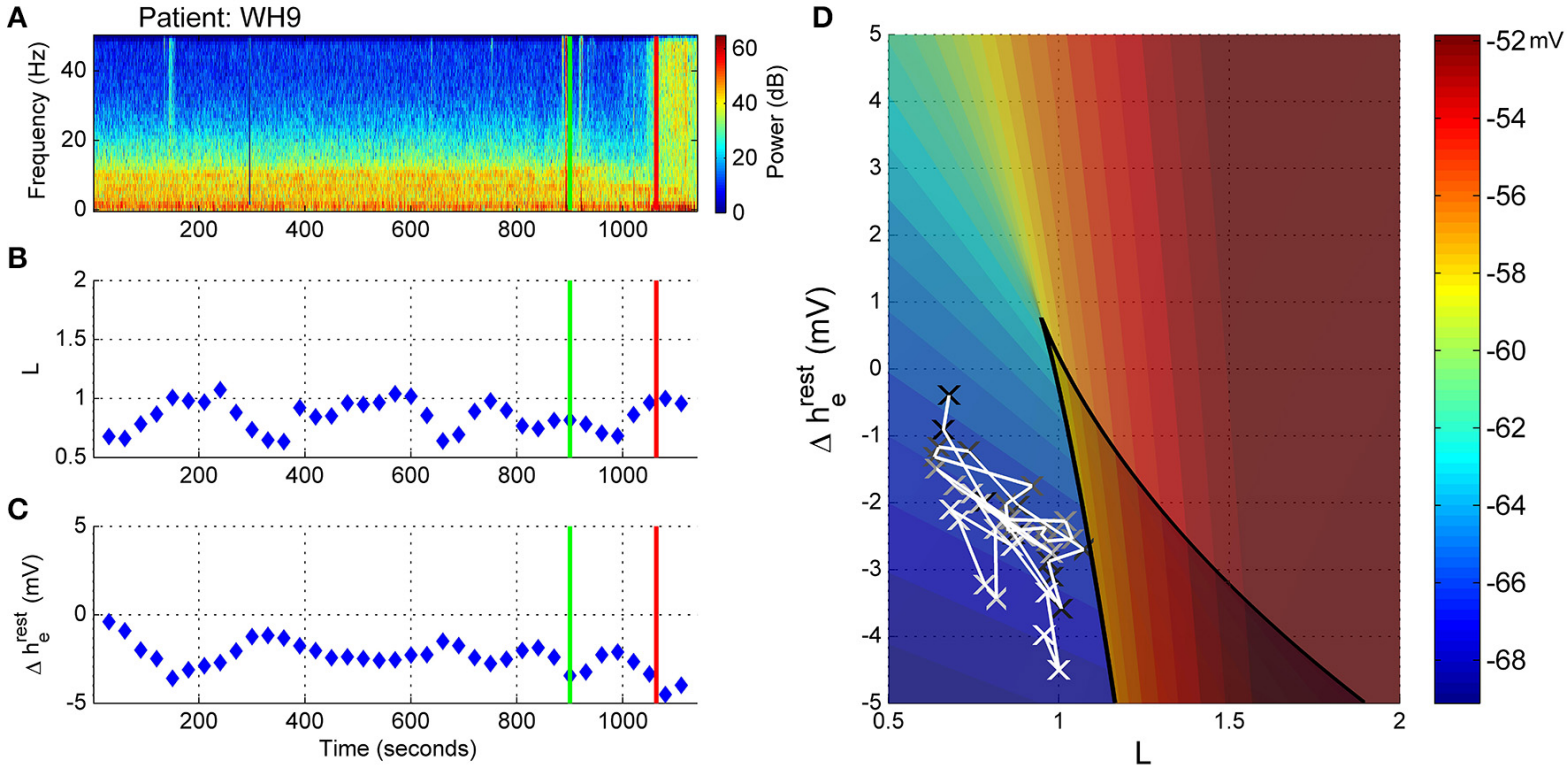
**Patient WH9, Sleep manifold. (A)** Spectrogram as in Figure 8. **(B)** Excitatory connection strength (L-parameter) over period of observation. **(C)** Change in resting membrane impedance (Δh^rest^_e_) over period of observation. **(D)** Resultant positioning on the sleep-manifold, with a black cross being the start of, and a white cross being the end of the observation period, with intermediate shades on the gray-scale representing the time progression between these time-points.

The trajectory in the state space reflected the lack of changes seen in the spectrogram. For the whole observation period, including the emergence period itself, the level of connection strength (L-parameter in Figure 9B) was generally low, while the resting impedance (Δh^rest^_e_ parameter in Figure 9C) showed a gradual decrease. We would conclude that there were no clear shifts in the emergence trajectory of the EEG in parameter space (Figure 9D), but rather this patient remained situated on the lower branch of the sleep-manifold for the entire emergence period. After ROR the apparent lack of increase in the excitatory connection strength is possibly caused by the ongoing strong delta and theta power that is dominating the spectrogram (see Figure 9A). It is likely to be due to muscle artifact, as evidenced by the sudden increase in broad-band high frequency power seen in the spectrogram.

### Patient WH57: non-archetypal emergence (no alpha, persistent delta, periods of high frequency activity)

**Figure 10:**
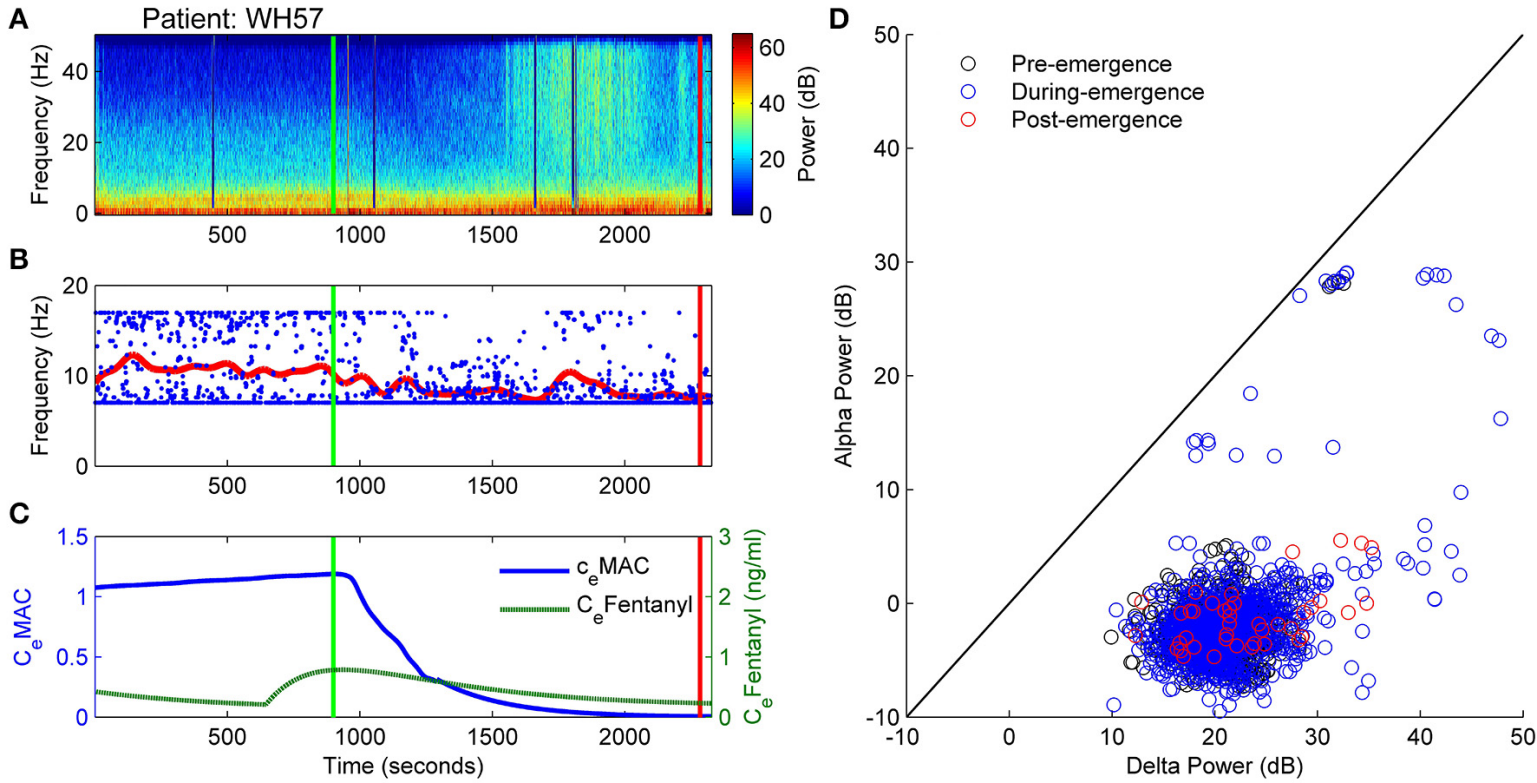
**Patient WH57: Non-archetypal emergence (no alpha, persistent delta, periods of high frequency activity). (A)** Spectrogram of the observation period. Start of emergence shown as a vertical green line at 900 s, time of patient response as a vertical red line. **(B)** Frequency of maximal oscillatory alpha power. **(C)** Concentration of anesthetic gas (C_e_MAC), blue line, left vertical axis, and opioid levels as equivalent Fentanyl (C_e_Fentanyl, ng/ml), green line, right vertical axis. (**D**) Absolute alpha power (dB) against absolute delta power (dB) for prior to start of emergence (black circles), during emergence period (blue circles), and following recovery of response (ROR).

In Figure 10 the EEG from an elderly patient shows a complete absence of alpha activity even during the maintenance phase of anesthesia. The frequency at maximal alpha peak in Figure 10B is purely artefactual, and jumping randomly between the 7 and 17Hz peak search limitation values. In the spectrogram (Figure 10A) episodes of high-frequency (20 to >50 Hz) activity are seen during emergence, indicated by the paler section between 1500 and 2000 s, the two dark-blue lines being recording artifact. Anesthetic concentrations were high for age adjusted MAC. C_e_Fentanyl levels ranged between 0.2 and 0.8 ng/ml (Figure 10C). The alpha and delta power levels remained at the same levels over the whole emergence process, the pre-emergence points (black circles) being obscured by the during- and post-emergence points at the same position (blue and red, Figure 10D).

**Figure 11:**
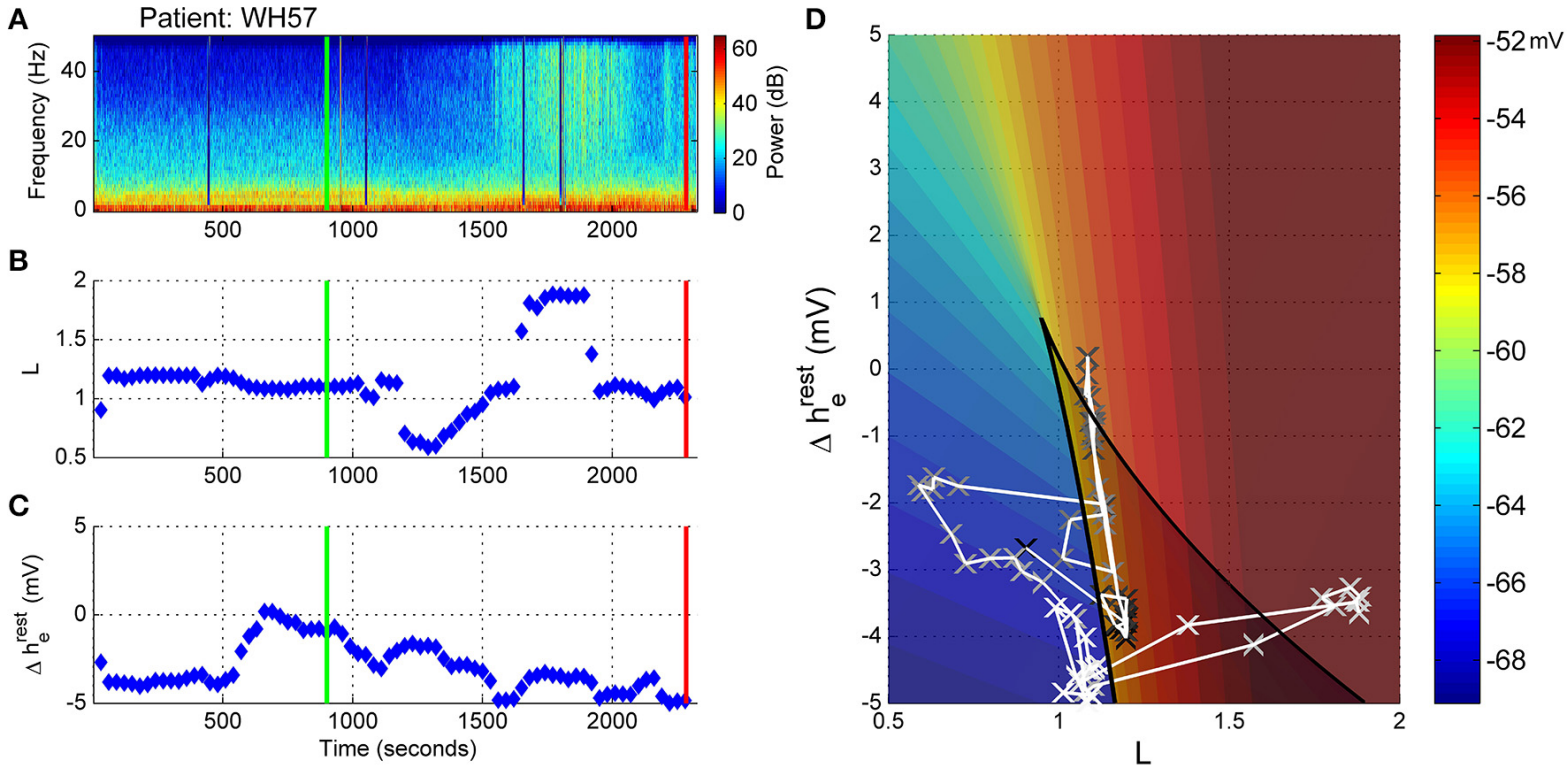
**Patient WH57, Sleep Manifold. (A)** Spectrogram as in Figure 10. **(B)** Excitatory connection strength (L-parameter) over period of observation. **(C)** Change in resting membrane impedance (Δh^rest^_e_) over period of observation. **(D)** Resultant positioning on the sleep-manifold, with a black cross being the start of, and a white cross being the end of the observation period, with intermediate shades on the gray-scale representing the time progression between these time-points.

Before the start of emergence there was a stable connection strength value of around 1.2 (Figure 11B). As the C_e_MAC decreased during the emergence period the connection strength initially decreased, but then increased (between 1600 and 2000 s), which corresponded to the period of high-frequency activity seen in Figure 11A. During this period the patient was flexing their arms to their head, but was not localizing and was not responsive to auditory commands in any way. The change in resting impedance (Δh^rest^_e_) climbs from −4 to 0 mV at 300 s prior to start of emergence, followed by a progressive decrease back to low levels over the emergence period.

On the sleep-manifold (Figure 11D) the most distinct finding was the excursion to, and return from, the top manifold of the state-space during the short period of increased connection strength. We infer, from the increase in L parameter and clinical state, that this patient had entered some pathological state of consciousness for about 10 min before falling back to unconsciousness and then becoming responsive.

### Dose response curves

**Figure 12:**
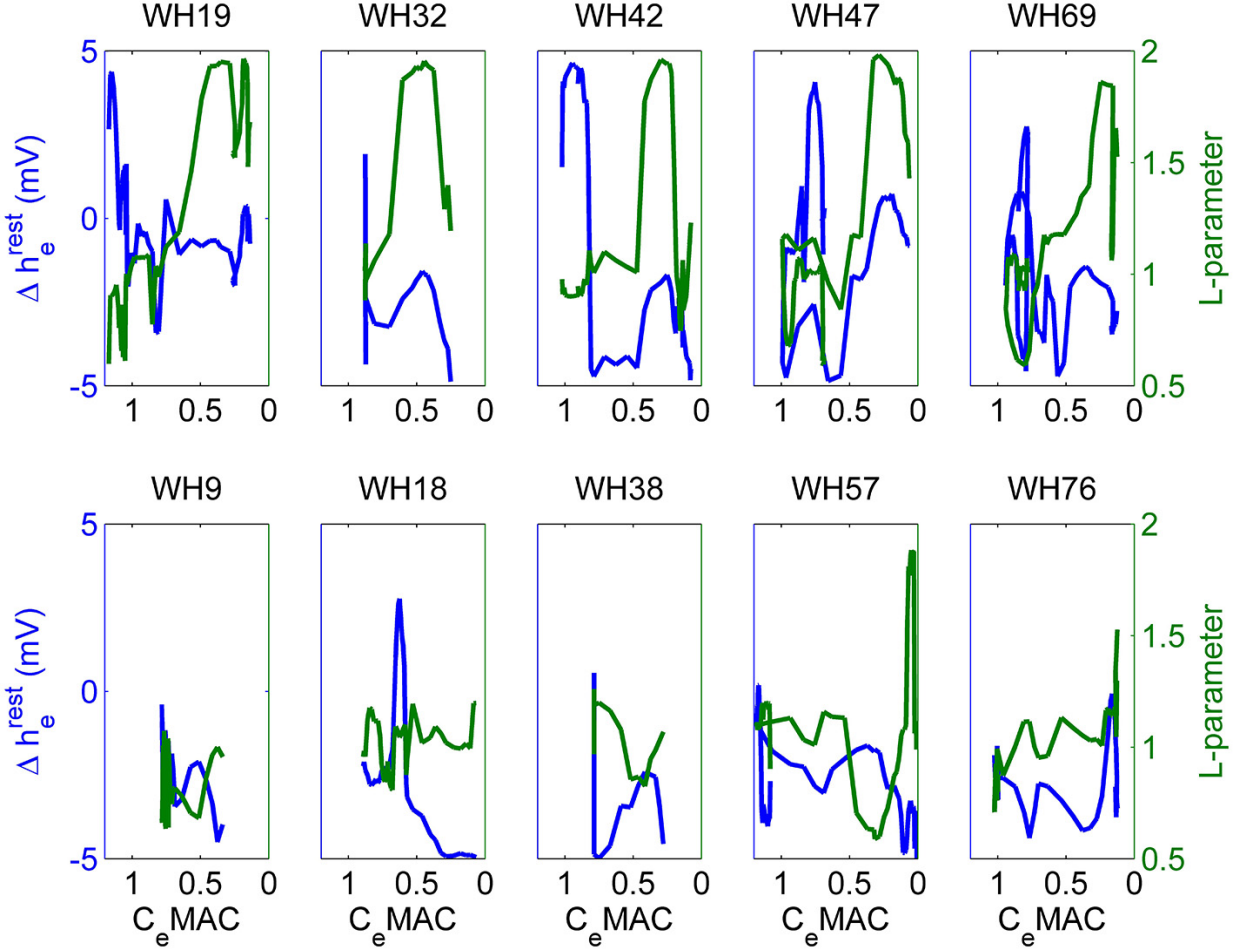
**Change in resting membrane impedance (Δh^rest^_e_, blue line, left axis), and excitatory connection strength (L-parameter, green line, right axis) plotted against decreasing anesthetic concentrations (C_e_MAC) for the entire observation period for 10 patients**. The five patients in the upper-half of the figure show “archetypal” emergences, those in the lower-half display “non-archetypal” emergences.

The somewhat perplexing differences in spectrogram and state-space trajectory, for the different patients, require some explanation. To examine the relationship between the anesthetic drugs and the EEG and state space parameters we plotted the dose response curves for C_e_MAC verses L and Δh^rest^_e_. We see that the five archetypal emergence patients (upper half of Figure 12) had very consistent patterns, consisting of an initial decrease in Δh^rest^_e_ occurring at around 0.8 MAC (blue line, left axis). (i.e., as surgery finishes, and often even before the anesthetic decreases much, they become hyperpolarized and move to the lower left region of the manifold). This seems to be a preliminary stage before a stereotypical pattern in late emergence when, at about 0.4 MAC, the L parameter (green line, right axis) suddenly increased for some time until abruptly decreasing again around 0.1 MAC; followed by the patient waking up a short time later. The non-archetypal patients (see lower half of Figure 12) showed much smaller changes in parameters—with a modest decrease in Δh^rest^_e_, and no change in L being the most consistent features.

## Discussion

At this early stage in developing the methodology, we are cautious in interpreting these results—and full quantitative analysis will require statistical evaluation of hundreds of case records. However we can conclude from our preliminary data that it is feasible to map features from the frontal EEG onto a state space of underlying biological parameters during emergence from general anesthesia. We also note that the changes observed in parameter values do not have an obvious direct correlation to simple observable features in the spectrogram. For example periods of high frequency activity do not consistently result in an increase in neuronal connection strength (L), and hence it would seem that the probabilistic mapping of multiple EEG features to the model appears to be a way of using the EEG to estimate changes of factors at a more abstract level than simply the obvious changes in frequency content of the EEG waveform itself. Model parameter and EEG feature choice will probably have to be further optimized for anesthesia, but we have at shown that intra- and inter-neuronal factors can behave independently. We view these results as part of an exploratory analysis, helping to determine which factors are relevant for further analysis with larger patient groups.

Patients who follow an archetypal emergence pattern seem to start with their cortex in a relatively low conductance state, and with poor cortical connection strength. They then follow quite a long trajectory in the state space before achieving the externally directed consciousness (the so-called “connected” consciousness) as described by Sanders et al. (2012). In contrast, patients who do not follow this archetypal emergence pattern typically have a hyperpolarized cortex for the whole observation period, irrespective of the level of anesthetic, and do not exhibit periods of high connection strength before the sudden engagement with the external environment. The fact that we have found many counterexamples to the archetypal pattern suggests that this pattern will not be completely reliable as an indicator of the causal biological processes that are necessary for the return of consciousness following general anesthesia and surgery.

It could be argued that—according to this model—a state of hyperpolarization (i.e., high neuronal conductance and low connectivity) is a prerequisite for the return of engaged consciousness as, for both groups, the transition to ROR took place from a hyperpolarized state—and that no patients transitioned to ROR from a depolarized state. The apparent hyperpolarization drift (occurring as the C_e_MAC *decreases* in emergence) seems to be opposite to the results of various animal studies using intra-neuronal recordings, which suggest that *increased* anesthesia is associated with some degree of neuronal *hyper*polarization. The whole methodology relies on the model having a reasonable fidelity to real physiology. There are two possibilities. Firstly, the model may be correct, and the animal experiments may be wrong, because they were conducted in the absence of surgical stimulation—which has a potent cortical depolarizing effect via aminergic activation. Or the model may be incorrect, and the observed hyperpolarization may be an artifact of the model stability. In essence, around the cusp of the fold on the manifold, the real parts of the eigenvalues for the system of equations are close to zero (or even positive depending on the parameter settings); and hence the steady-states of the model show marginal stability. This is manifest in the EEG as maximal delta and alpha oscillations in this region (see Figure 11 in Dadok et al., 2014). As the surgical stimulation subsides toward the end of the operation, and the C_e_MAC starts to decrease, the mean delta gradient decreases and the state of the cortex as represented on the manifold moves downwards away from the cusp to a more stable state. In fact the idea of delta waves as a sign of a very hyperpolarized thalamo-cortical system is over-simplistic. For example in the well-described phenomenon of “delta-arousal,” there is an *increase* in delta power associated with increased surgical noxious stimulation (Morimoto et al., 2005). It is possible that we are seeing the opposite phenomenon—a decrease in painful stimulation resulting in a decrease in delta power. There also are other data that suggest that large amplitude EEG is a sign of excessive noxious stimulation or inadequate analgesic medication (Liley et al., 2010) or even nitrous oxide withdrawal (Foster and Liley, 2011).

A characteristic feature of the archetypal patients is the consistent increase in the L-parameter for a period prior to ROR. It is tempting to associate these episodes with the first forays into consciousness, although this would have to be described as a dissociated consciousness, i.e., not engaged with the outside world, indicative of something like dreaming, as these patients were still unresponsive at that time. Yet given that only one group displayed these episodes of increased connection strength we would have to conclude that, assuming a well-functioning model, either connection strength is irrelevant to engaged (externally directed) consciousness, or that the mechanisms required for engaged consciousness are hidden from the model. For both groups discontinuous, abrupt changes were seen in brain dynamics. For the archetypal patients the changes in L were abrupt, not gradual progressive changes; and for the non-archetypal patients the ROR was not preceded by any indicators in the spectrogram. The model has all patients positioned in the left lower corner of manifold prior to ROR. The important feature of this region is that it is near to the 3-root area of the manifold; an area of instability where a small change in parameter value results in discontinuous transitions in state. We speculate that the reason all patients either migrated to, or were already present in this area prior to ROR is that this would make it much easier for the brain to transition to another state. In contrast, if patients showing the archetypal pattern of emergence remained at a depolarized state, a small change in parameter would not lead to a large change in state given the gradient. It is much harder for the cortex to climb than to jump. This may help to explain the “flip-flop” phenomena that have been described in the natural sleep literature (Saper et al., 2001).

The above argument only holds if one assumes the true position for the wakeful cortex is on the higher branch of the manifold; in our data this has been obscured by the presence of broadband EMG, or it may have been overlooked due to a very short lasting ROR, e.g., 5 s, prior to a return to some sedation; this would not show on the model which requires 30 s sections of EEG. These results are provisional, and there are some significant issues still to be resolved. Our EEG data were collected from a single pre-frontal channel, and hence completely lacking in spatial information. We also note that the “sleep” model is in some respects incomplete when applied to general anesthesia, because it does not produce burst suppression patterns, various high frequency oscillations, and does not distinguish between the dissociated consciousness of REM sleep and true wakeful consciousness.

## Conclusions

The archetypal EEG pattern of emergence is not the only pattern of emergence seen in surgical patients, with many patients showing no obvious progressive changes in their EEG until sudden recovery of responsiveness. When the EEG features are mapped onto a model state space of cortical connection strength and intrinsic resting neuronal conductivity, patients consistently show a low level of excitatory connectedness during anesthesia. During emergence the archetypal patients show a very consistent trajectory of progressive decrease in neuronal impedance and sudden increase in connection strength before waking. In contrast, the non-archetypal patients showed minimal changes in either parameter before waking. We therefore conclude that the archetypal EEG emergence pattern is not a necessary prelude to recovery of responsiveness; and hence is probably an epiphenomenon as regards our understanding of the mechanisms and signs of anesthetic-induced unconsciousness.

### Conflict of interest statement

The authors declare that the research was conducted in the absence of any commercial or financial relationships that could be construed as a potential conflict of interest.
